# Supplementary material for: Let-7 microRNA-dependent control of leukotriene signaling regulates the transition of hematopoietic niche in mice
Source: Nat Commun. 2017 Jul 25;8:128. doi: 10.1038/s41467-017-00137-y (PMC5527007; doi:10.1038/s41467-017-00137-y)
Supplement: Supplementary file 2 — Supplementary Information [file 41467_2017_137_MOESM2_ESM.pdf]

Title of file for HTML: Peer Review File
Description:

Title of file for HTML: Supplementary Information
Description: Supplementary Figures, Supplementary Tables.

Title of file for HTML: Supplementary Data 1
Description: The next-generation sequencing was performed using RNAs from ECs (CD31+ Kit- CD45-) or HSPCs (CD31+ Kit+ CD45-) from E11.5 Ctr or cKO embryos. Results are shown as average RPKM (reads per kilobase per million mapped reads).

Title of file for HTML: Supplementary Data 2
Description: Small RNA sequencing (Illumina) was performed using RNAs from CD31+ cells derived from E11.0 AGM in cKO or Ctr embryos. Sequence was performed in triplicates and the list contains miRNAs which are differentially expressed in cKO vs Ctr with P

Title of file for HTML: Supplementary Movie 1
Description: Ctr E11.5 embryo shows heartbeat.

Title of file for HTML: Supplementary Movie 2
Description: cKO E11.5 embryo shows heartbeat.

Title of file for HTML: Supplementary Movie 3
Description: Blood flow in the yolk sac of E11.5 Ctr embryos.

Title of file for HTML: Supplementary Movie 4
Description: Blood flow in the yolk sac of E11.5 cKO embryos.

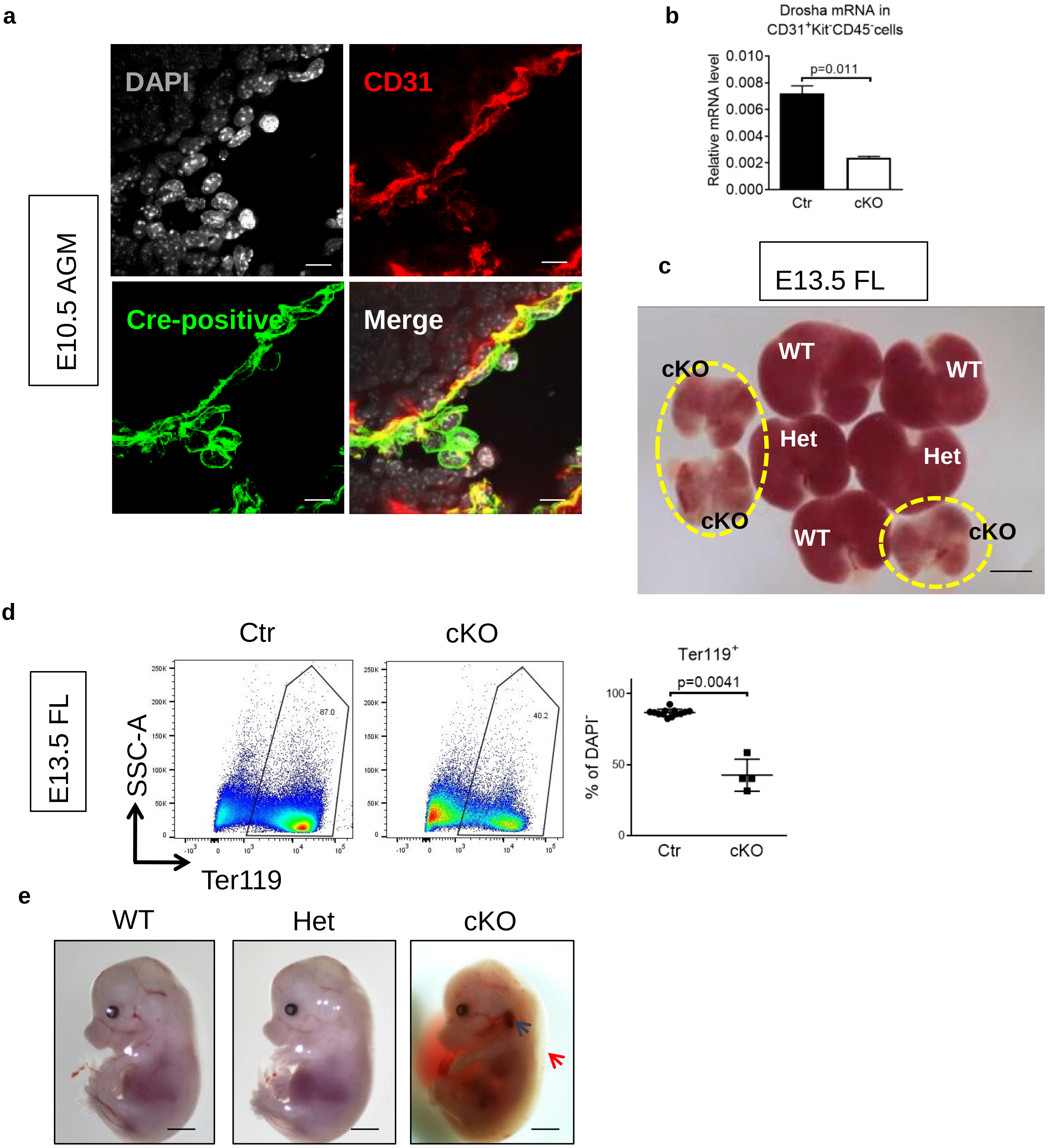

Supplementary Figure 1 Excision of *Drosha* by Cdh5-Cre occurs in the endothelium. a. Representative images of immunofluorescence staining of transverse sections of E10.5 AGM. Endothelial cells (red, $CD31^+$) and Cre-positive cells (green) were stained with anti-CD31 antibody and anti-GFP antibody, respectively. Scale bar: 10 µm. **b.** qRT-PCR analysis of *Drosha* mRNAs in endothelial cells ($CD31^+Kit^-CD45^-$) from E11.5 Ctr or cKO embryos. Results were shown as Mean±SEM. P-values were generated by unpaired student t-test. n=Ctr: 12 embryos, cKO: 6 embryos. 2 litters. **c.** Representative images of fetal livers of E13.5 Ctr (WT and Het) and cKO embryos (yellow circles) from the same litter. Scale bars: 2mm. **d.** Flow cytometric analysis of erythroid lineage cells by measuring percentage of $Ter119^+$ cells among live cells ($DAPI^-$) in Ctr and cKO E13.5 fetal livers. Right panels shows quantification as Mean±SEM. P-values were generated by unpaired student t-test. n=Ctr: 13 embryos, cKO: 4 embryos. 3 litters. **e.** Representative images of gross morphology of E14.5 Ctr and cKO embryos. Red and blue

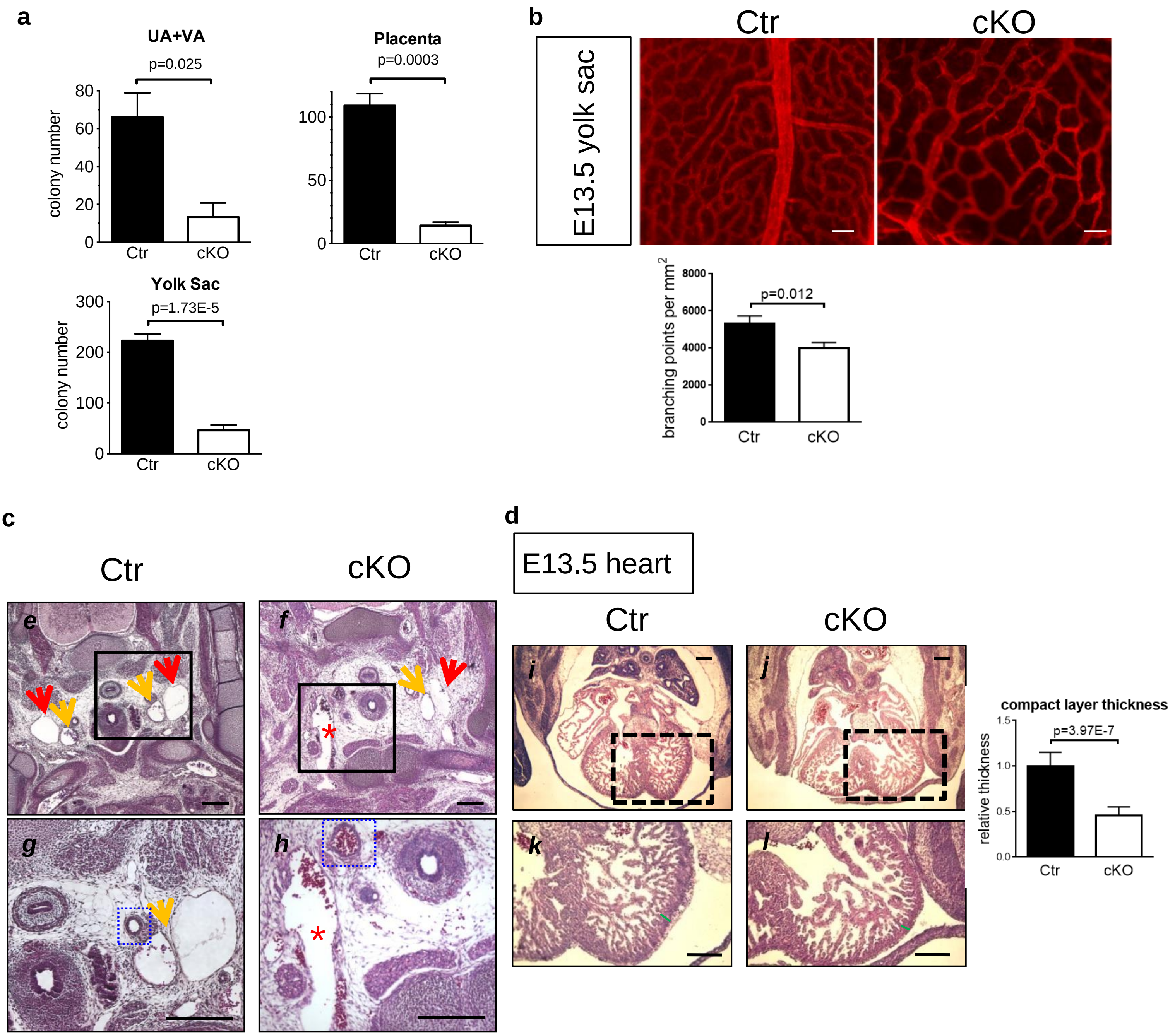

Supplementary Figure 2 *Drosha* cKO embryos exhibit mild developmental defects. a. All the cells from E11.5 Ctr and cKO umbilical and vitelline arteries (UA+VA) were subjected to CFU assay. Ten percent of the cells from E11.5 Ctr and cKO placenta were subjected to CFU assay. All the cells from E9.5 Ctr or cKO yolk sac were subjected to CFU assay. Total colony counts of 3 different progenitors (BFU-E, CFU-GM, and CFU-GEMM) were plotted as Mean$\pm$SEM. P-values were generated by unpaired student t-test. For UA+VA and placenta, n=Ctr: 6 embryos, cKO: 3 embryos. 1 litter. For yolk sac, n=Ctr: 5 embryos, cKO: 4 embryos. 1 litter. **b.** Whole mount immunofluorescence staining of CD31 (top) and quantification of branching points of the vasculature in the yolk sac of Ctr and cKO embryos (bottom). Results were plotted as Mean±SEM. P-values were generated by unpaired student t-test. n=Ctr: 4 embryos, cKO: 5 embryos. 2 litters. Scale bars: 10 μm. **c.** H&E staining of transverse thoracic sections of Ctr and cKO embryos. Areas indicated with a black box in (e) and (f) were magnified and presented in (g) and (h). Yellow arrows: internal jugular vein; Red arrows: jugular lymph sac; blue boxes: carotid artery; red asterisk: fused lymph sac and jugular vein. Scale bars: 100 μm. **d.** Representative images of transverse sections of E13.5 embryonic heart from Ctr (*i*, and *k*) and cKO embryos (*j* and *l*). Areas indicated with a black box in upper panels (***i*** and ***j***) were magnified and presented in lower panels (*k* and *l*). Green lines indicate the thickness of the compact layer of the left ventricle. Thickness of left ventricle compact layer relative to Ctr is shown (right) as Mean$\pm$SEM. P-values were generated by unpaired student t-test. n=Ctr: 3 embryos, cKO: 3 embryos. 2 litters. Scale bars: 100 μm.

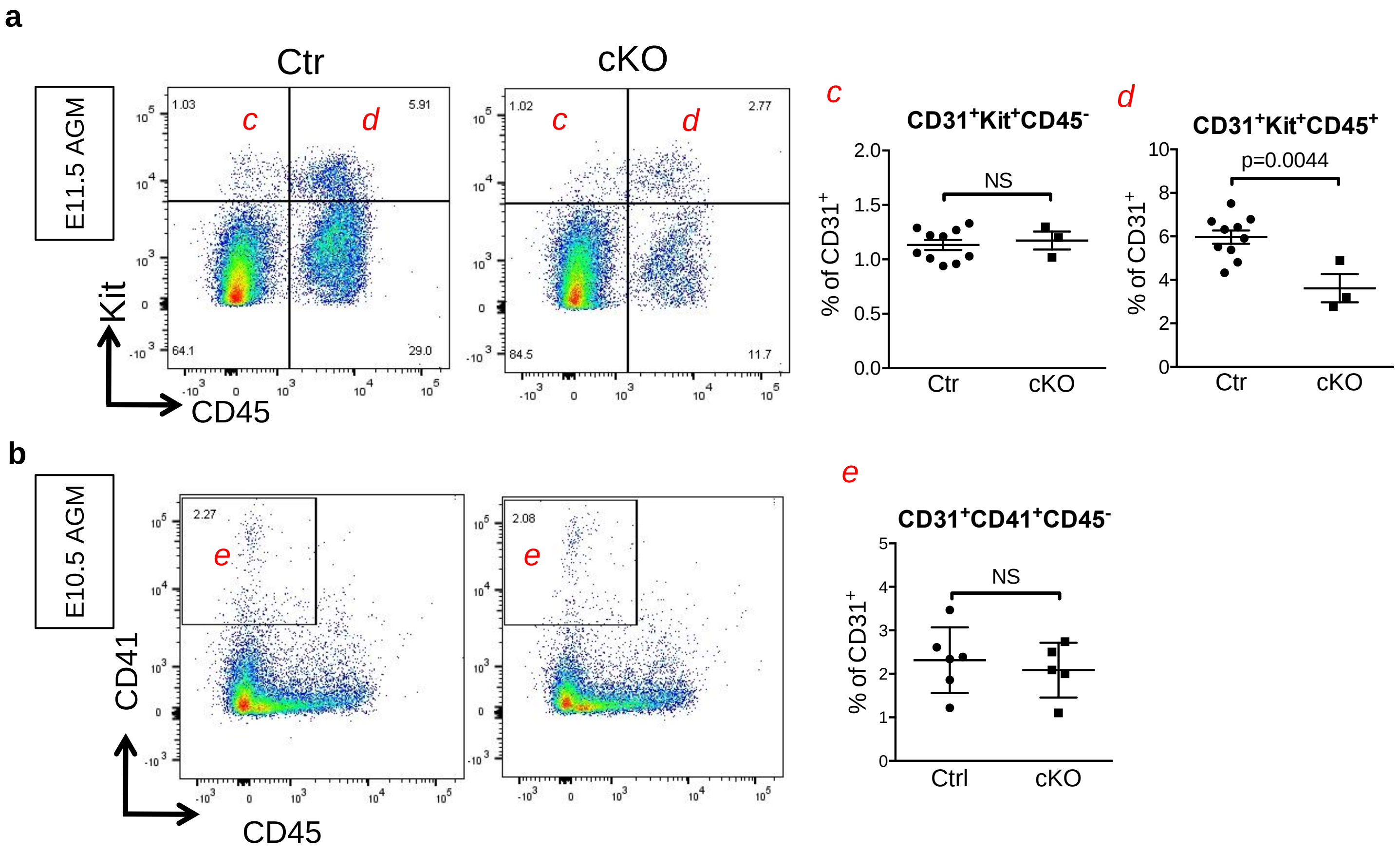

Supplementary Figure 3 AGMs from cKO embryos contain HSPC clusters comparable to control embryos.

a. Flow cytometric analysis of the fraction (%) of HSPC clusters (c, $CD31^+Kit^+CD45^-$) and maturing HSPCs (d, $CD31^+Kit^+CD45^+$) among $CD31^+$cells was performed in E11.5 Ctr or cKO AGMs and plotted as Mean±SEM. P-values were generated by unpaired student t-test. n=Ctr: 12 embryos, cKO: 3 embryos. 2 litters. **b.** Flow cytometric analysis of the fraction (%) of HSPC clusters ($CD31^+CD41^+CD45^-$) among $CD31^+$cells were performed in E10.5 Ctr or cKO AGMs and plotted as Mean±SEM. P-values were generated by unpaired student t-test. n=Ctr: 11 embryos, cKO: 6 embryos. 4 litters. NS, not significant.

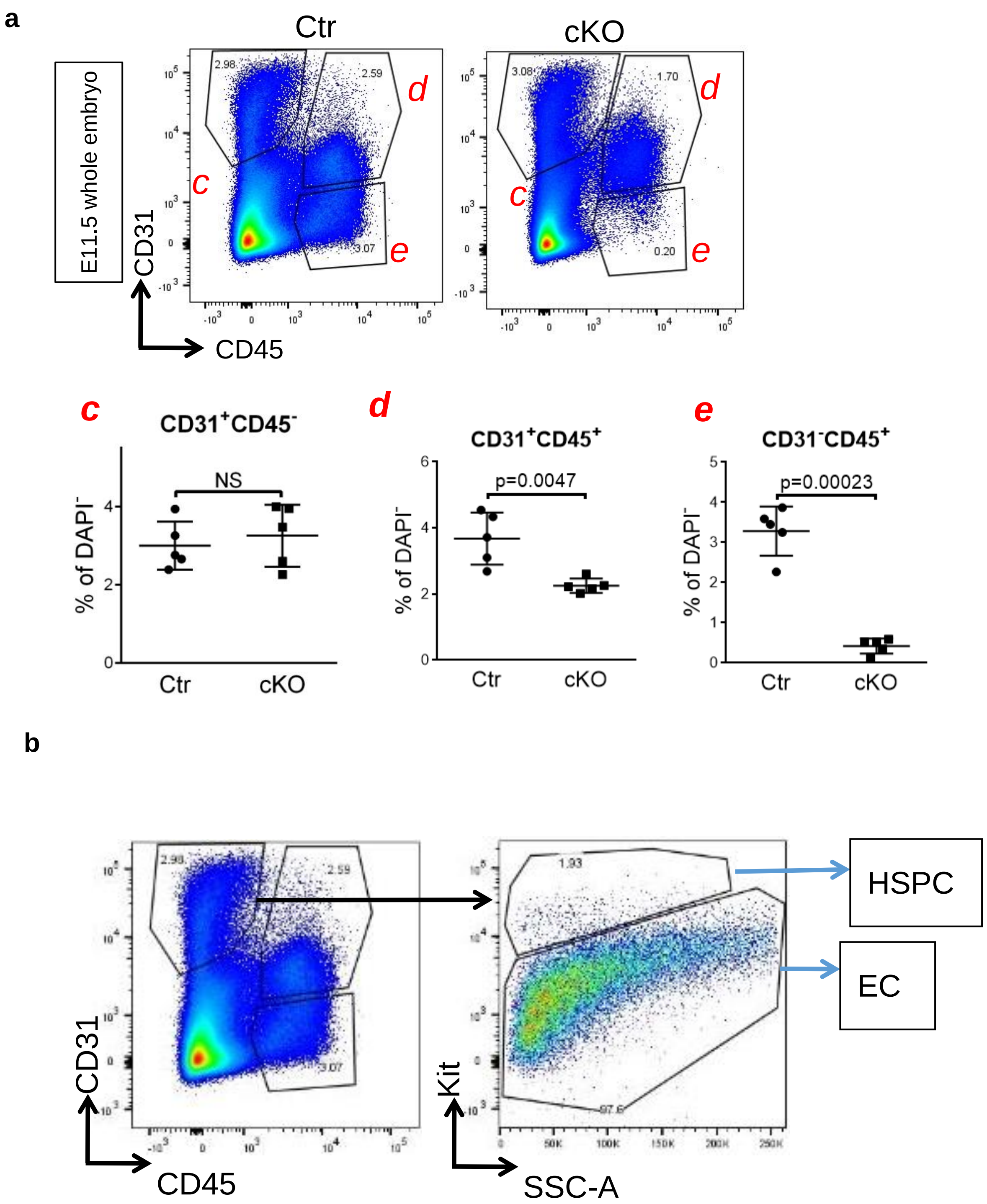

Supplementary Figure 4 HSPC clusters and ECs are sorted for RNA seq. a. Flow cytometric analysis of the fraction (%) of ECs ($CD31^+CD45^-$), mature HSPCs ($CD31^+CD45^+$), and mature hematopoietic cells ($CD31^-CD45^+$) among live cells ($DAPI^-$) was performed in E11.5 Ctr or cKO whole embryos and plotted as Mean±SEM. P-values were generated by unpaired student t-test. n=Ctr: 5 embryos, cKO: 5 embryos. 4 litters. **b**. Schematic diagram of cells subjected to the next generation RNA and small RNA sequencing. HSPC cluster cells ($CD31^+Kit^+CD45^-$) and ECs ($CD31^+Kit^-CD45^-$) of whole E11.5 embryos were sorted for next-generation RNA sequencing. n=Ctr: 13 embryos, cKO: 5 embryos. 3 litters. Embryos of same geneotype were pooled. NS, not significant.

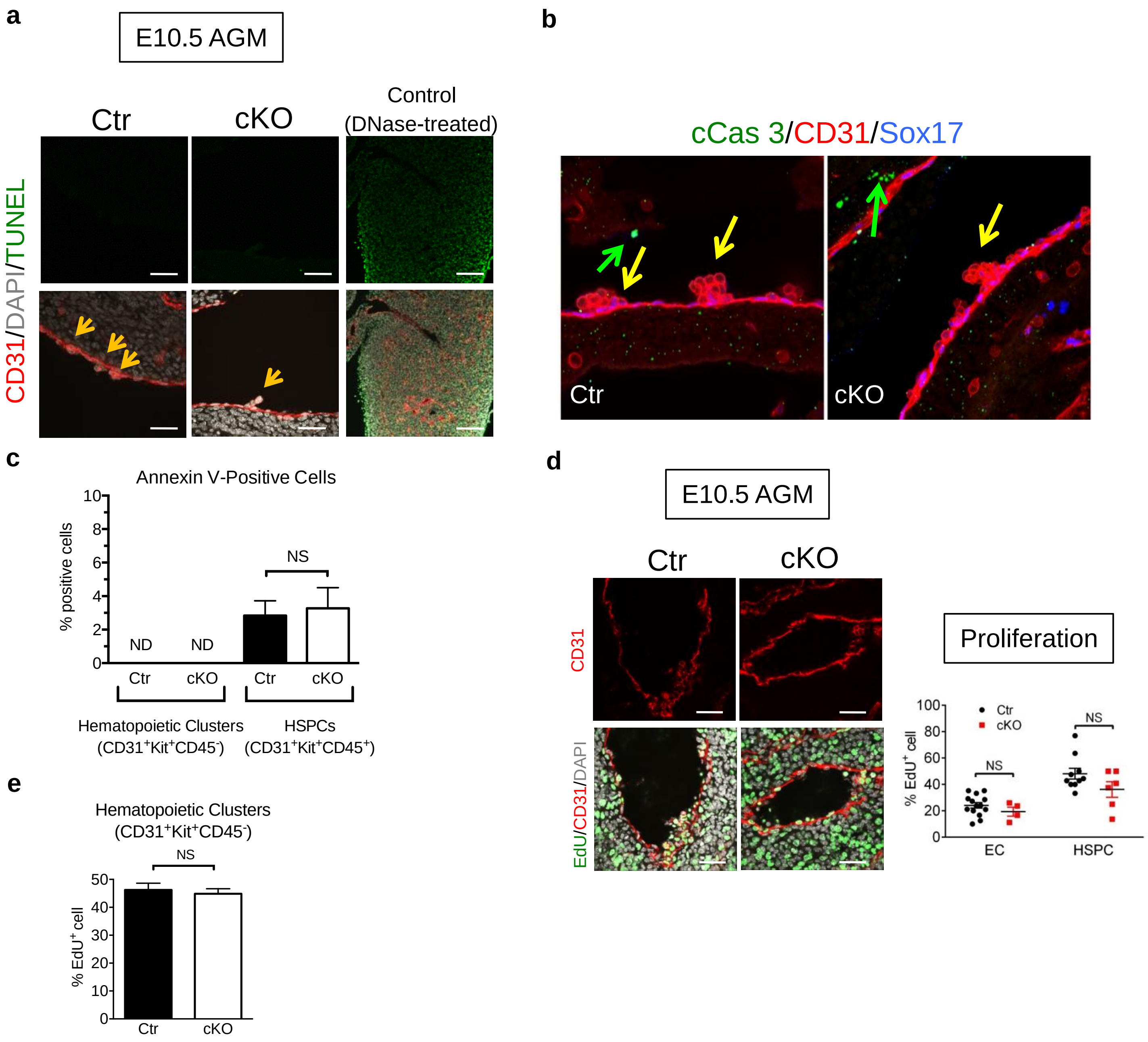

Supplementary Figure 5 Premature cell death or a proliferation abnormality cannot be found in intra-aortic clusters in Drosha mutant mice. a. TUNEL assay was performed on the transverse section of E10.5 Ctr and cKO dorsal aorta (DA). Arrows indicate HSPC clusters. Sections of DA were treated with DNase I as positive control (right). n=Ctr: 3 embryos, cKO: 3 embryos. 2 litters. Scale bars: 50 μm. **b.** Whole mount immunofluorescence staining of CD31 (red), Sox17 (blue), and cleaved-Caspase 3 (cCas 3, green) of AGMs from E10.5 cKO (right) or Ctr embryos (left). n=Ctr: 3 embryos, cKO 3 embryos. 2 litters. Yellow and green arrows indicate cluster cells and apoptotic (cCas 3-positive) cells, respectively. Scale bars: 50 μm. **c.** The percentage of apoptotic cells among hematopoietic clusters ($CD31^+Kit^+CD45^-$) or HSPCs ($CD31^+Kit^+CD45^+$) from E10.5 Ctr or cKO AGM was quantitated by flow cytometric analysis of Annexin V-positive cells. There were no Annexin V-positive cells in the hematopoietic clusters, indicated as ND (not detected). Around 3% apoptotic cells (Annexin V-positive cells) were detected in HSPCs from Ctr and cKO. P-values were generated by unpaired student t-test. **d.** Proliferation rate was measure by injecting EdU inraperitoneally to E10.5 pregnant female mice. After 2 hours, embryos were harvested and cryosectioned at a thickness of 50 mm. Representative images of EdU stain and immunofluorescence stain of CD31 were shown. The fraction (%) of S-phase (EdU^+) cells in endothelial cells (EC) and HSPC cluster cells (HSPC) were plotted as Mean±SEM. P-values were generated by unpaired student t-test. n=Ctr: 2 embryos, cKO: 2 embryos. 1 litter. Scale bars: 50 μm. **e.** The percentage fraction of S-phase (EdU positive) cells among hematopoietic clusters ($CD31^+Kit^+CD45^-$) from E10.5 Ctr or cKO AGM was quantitated by flow cytomeric analysis. P-values were generated by unpaired student t-test. Embryos were harvested 1 hour after intraperitoneal injection of EdU (50 mg/Kg). n=Ctr: 10 embryos, cKO: 5 embryos. 3 litters.

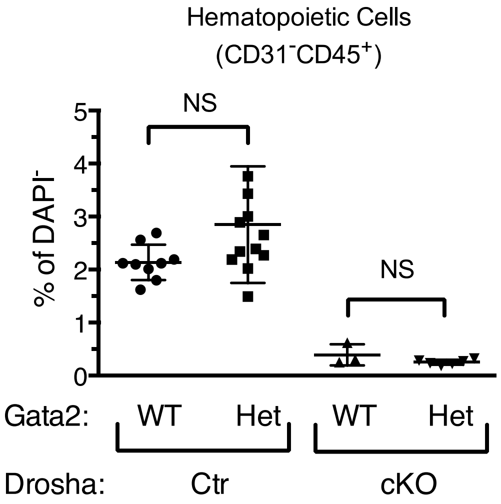

Supplementary Figure 6 Haploinsufficiency of *Gata2* in the endothelium of Drosha mutants does not rescue hematopoietic defects. Flow cytometric analysis of the faction (%) of hematopoietic cells (CD31$^-$CD45$^+$) per total live (DAPI$^-$) cells in E11.5 AGMs from *Drosha* Ctr;*Gata2* WT, *Drosha* Ctr;*Gata2* Het, *Drosha* cKO;*Alox5* WT or *Drosha* cKO;*Alox5* Het embryos. *Gata2* Het is *Gata2*$^{fl/+}$; *Cdh5*-Cre$^+$. Results were plotted as Mean±SEM. P-values were generated by unpaired student t-test. *Drosha* Ctr;*Gata2* WT n=9 embryos. *Drosha* Ctr;*Gata2* Het n=11 embryos. *Drosha* cKO;*Gata2* WT n=3 embryos. *Drosha* cKO;*Gata2* Het n=6 embryos. 5 litters. NS, not significant.

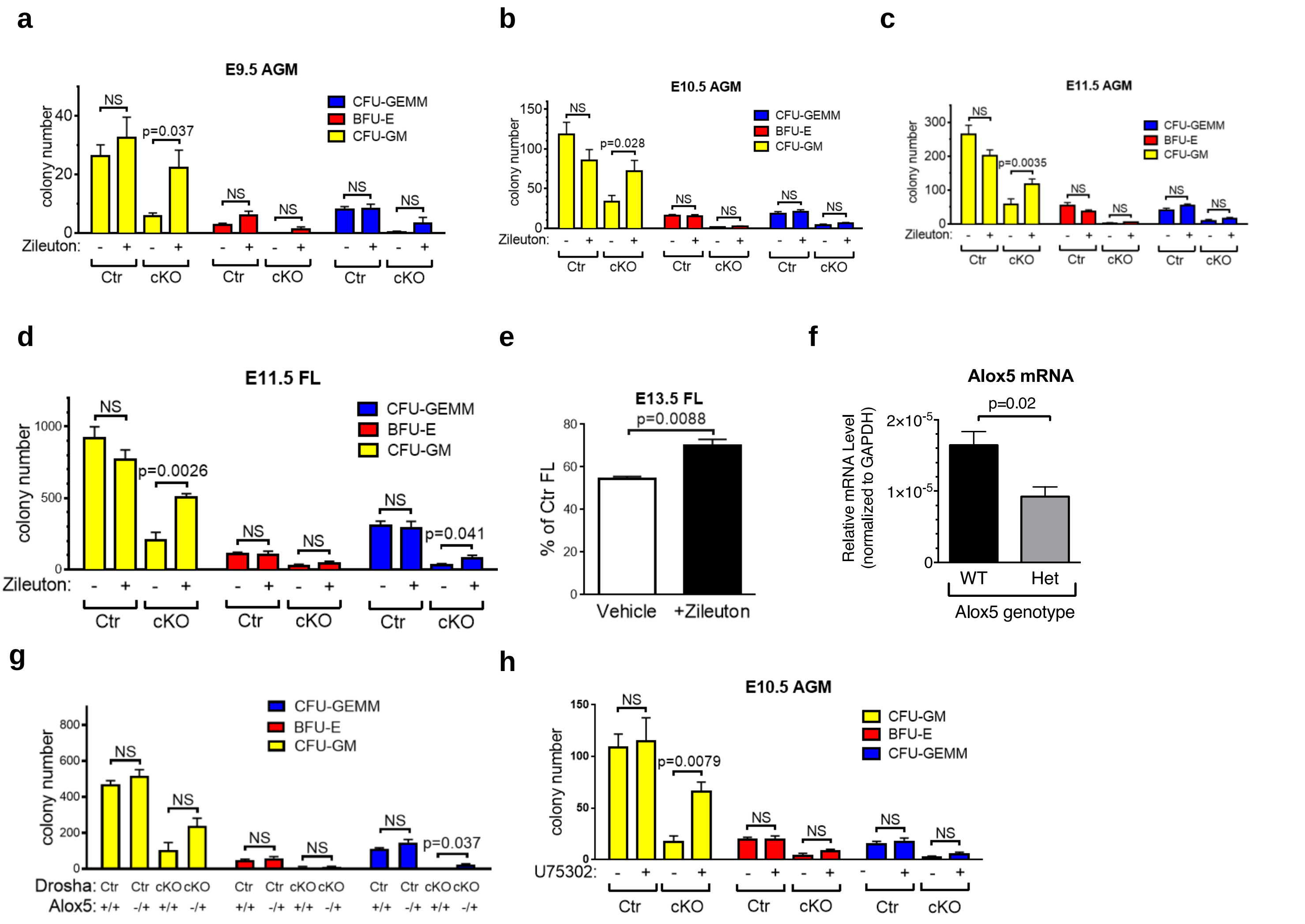

Supplementary Figure 7 Inhibition of the Alox5/LTB4/BLT1 pathway rescued the hematopoietic defects of *Drosha* cKO embryos. a. and b. AGMs harvested from E9.5 (**a**) or E10.5 (**b**) Ctr or cKO embryos were incubated with 50 nM zileuton or vehicle (50%DMSO/50%PBS) overnight and subjected to CFU assay. Colony counts were plotted as Mean±SEM. At E9.5, n=Ctr: 11 embryos, cKO: 6 embryos. 3 litters for zileuton. , n=Ctr: 11 embryos, cKO: 3 embryos. 3 litters for vehicle. At E10.5. , n=Ctr: 18 embryos, cKO: 9 embryos, 5 litters for zileuton. n=Ctr: 18 embryos, cKO: 10 embryos, 6 litters for vehicle. P-values were generated by unpaired student t-test. **c.** and **d.** Five mg/kg zileuton or vehicle was injected into pregnant mice at E9.5 and E10.5. At E11.5, AGM and fetal liver were harvested and subjected to CFU assay. Twenty % of cells from AGM (**c**) and 10% of the cells from fetal liver (**d**) were used. Colony counts were plotted as Mean±SEM. For vehicle, n=Ctr: 10 embryos, cKO: 5 embryos. 3 litters. For zileuton, n=Ctr: 11 embryos, cKO: 6 embryos. 4 litters. P-values were generated by unpaired student t-test. **e.** The size of E13.5 fetal liver (FL) from vehicle or zileuton injected litters was quantitated by ImageJ. The size of cKO-FL relative to Ctr-FL size was plotted as Mean±SEM. For zileuton, n=Ctr: 3 embryos, cKO: 3 embryos. 1 litter. For vehicle, n=Ctr: 5 embryos, cKO: 3 embryos. 1 litter. P-values were generated by unpaired student t-test. **f.** Total RNAs were isolated from E10.5 embryos, followed by qRT-PCR analysis of Alox5 mRNA and plotted as Mean±SEM. P-values were generated by unpaired student t-test. *Alox5*+/+ n=4 embryos, *Alox5*+/- n=5 embryos. 2 litters. **g.** E13.5 FLs were subjected to CFU assay. Number of colonies were plotted as Mean±SEM. P-values were generated by unpaired student t-test. *Drosha* Ctr;*Alox5*$^{+/+}$ n=9 embryos, *Drosha* Ctr;Alox5$^{+/-}$ n=8 embryos, *Drosha* cKO;Alox5$^{+/+}$ n=3 embryos, and *Drosha* cKO; Alox5$^{+/-}$ n=4 embryos. 5 litters. **h.** AGMs from E10.5 Ctr or cKO embryos were incubated with 2.5 μM U75302 or vehicle (ethanol) overnight and then subjected to CFU assay. Colony counts were plotted as Mean±SEM. P-values were generated by unpaired student t-test. For U75302 treatment, n=Ctr: 5 embryos, cKO: 4 embryos. n=3 litters. For vehicle treatment, n=Ctr: 7 embryos, cKO: 4 embryos. 2 litters. NS, not significant.

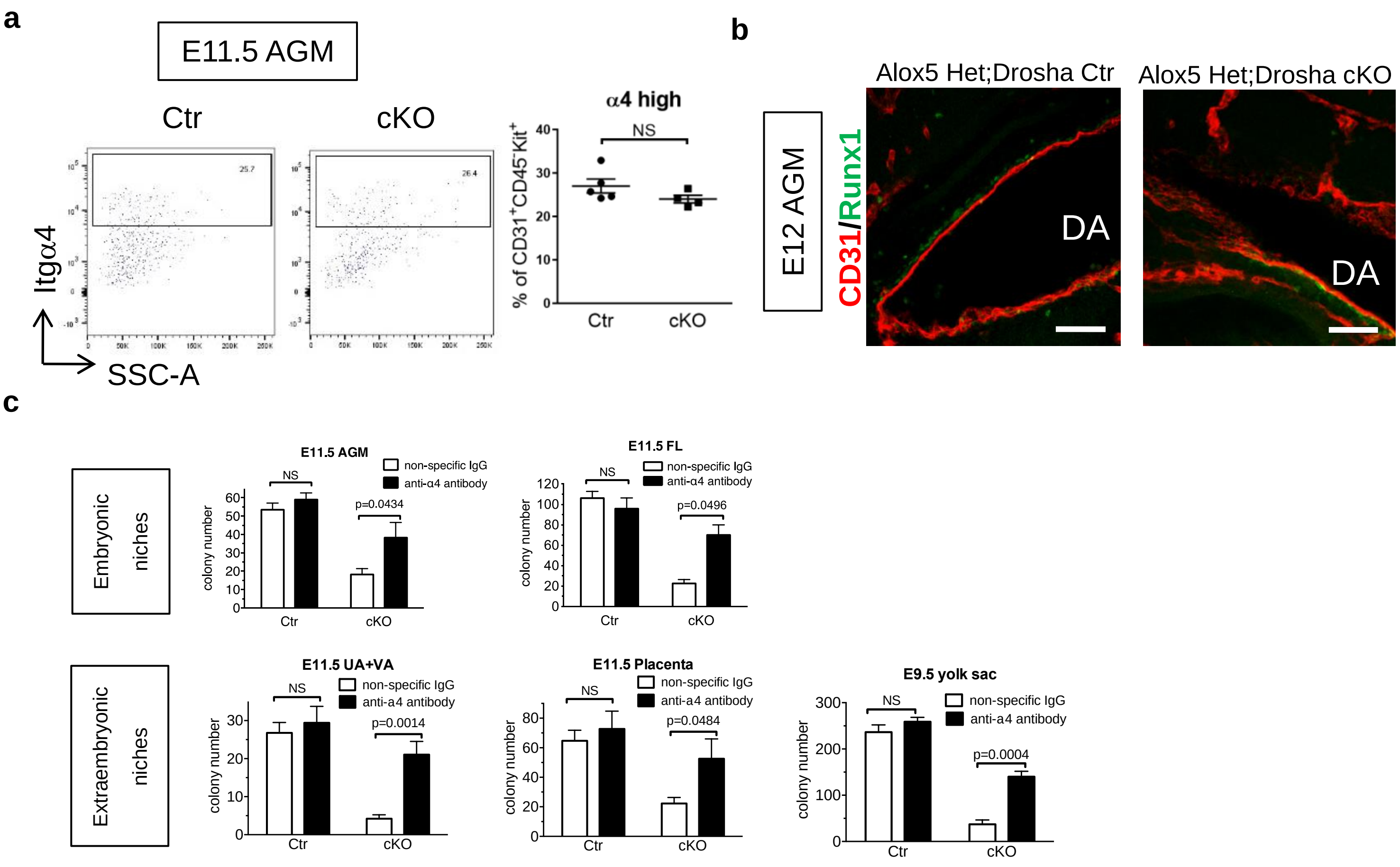

Supplementary Figure 8 Inhibition of Alox5/LTB4/Itgα4 pathway rescued the hematopoietic defects of *Drosha* cKO embryos. a. Flow cytometric analysis of a fraction (%) of Itgα4 high population among HSPC clusters ($CD31^+CD45^-Kit^+$) from AGMs of E11.5 Ctr or cKO embryos. Result was plotted as Mean±SEM. P-values were generated by unpaired student t-test. n=Ctr: 12 embryos, cKO: 3 embryos. 3 litters. **b.** Whole mount immunofluorescence stain of Runx1 (green) and CD31 (red) with E12.0 AGM of the indicated genotype. Alox5 Het:Drosha Ctr, n=4 embryos, Alox5 Het:Drosha cKO, n=2 embryos, 2 litters. **c.** Anti-Itgα4 antibody or non-specific IgG (control) was injected into pregnant mice, followed by CFU assay using cells harvested from embryonic or extraembryonic niches of HSPCs of Ctr or cKO embryos. For AGMs, fetal livers, placenta and umbilical and vitelline arteries (UA+VA), control IgG group includes n=12 Ctr embryos, 5 cKO embryos. 3 litters. Anti-Itga4 antibody group includes n=7 Ctr embryos, 4 cKO embryos. 2 litters. For yolk sac, control IgG group includes n=18 Ctr embryos, 4 cKO embryos. 3 litters. Anti-Itgα4 antibody group includes n=5 Ctr embryos, 5 cKO embryos. 1 litter. Total counts of colonies (BFU-E, CFU-GM, and CFU-GEMM) were plotted as Mean±SEM. P-values were generated by unpaired student t-test. NS: not significant.

		Total Number of Colonies			Percentage of Colonies		
Sample	Genotype	**Δ/ Δ**	**Δ/ f**	**f/f**	**Δ/ Δ**	**Δ/ f**	**f/f**
E10.5 AGM	*Drosha*$^{fl/fl}$; *Cdh5-Cre*$^{+}$	0	154	42	0	79	21

Supplementary Table 1 Excision of the *Drosha* flox allele in colonies derived from E10.5 cKO AGM. E10.5 AGM from cKO or Ctr embryos were subjected to CFU assay. Colonies from E10.5 AGM of cKO embryos were picked up and genotyped. Δ stands for excised allele. f stands for floxed allele.

	Drosha$^{fl/+}$		***Drosha***$^{fl/fl}$		***Drosha***$^{fl/+}$**;** ***Cdh5-cre***$^{+}$		***Drosha***$^{fl/fl}$**;** ***Cdh5-cre***$^{+}$	
	EP	AP	EP	AP	EP	AP	EP	AP
E13.5 (6 litters)	25%	12(19.4%)	25%	17(27.4%)	25%	18(29.0%)	25%	15(24.2%)
E14.5 (3 litters)	25%	8(36.3%)	25%	5(22.7%)	25%	5(22.7%)	25%	4(18.2%)
E15.5 (5 litters)	25%	8(24.4%)	25%	10(30.3%)	25%	14(42.4%)	25%	1(3.0%)
E16.5 (3 litters)	25%	5(25.0%)	25%	8(40.0%)	25%	7(35.0%)	25%	0(0%)

Supplementary Table 2 Genotyping analysis showed Drosha cKO embryos die between E14.5 and E15.5.Yolk sac is dissected from live embryos, whose heart is still beating, for genotyping. Expected ratios of *Drosha*$^{fl/+}$, *Drosha*$^{fl/fl}$, *Drosha*$^{fl/+}$;*Chd5-Cre*$^{+}$ and *Drosha*$^{fl/fl}$;*Cdh5-cre*$^{+}$ are ¼, ¼, ¼ and ¼. EP: expected ratio. AP: actual ratio.

		Total Number of Colonies			Percentage of Colonies		
Sample	Genotype	**Δ/ Δ**	**Δ/ f**	**f/f**	**Δ/ Δ**	**Δ/ f**	**f/f**
E11.5 FL	*Drosha*$^{fl/fl}$; *Cdh5-Cre*$^{+}$	0	12	1	0	92	8
E11.5 AGM	*Drosha*$^{fl/fl}$; *Cdh5-Cre*$^{+}$	0	3	1	0	75	25

Supplementary Table 3 Excision of the *Drosha* flox allele in colonies derived from sorted CD45+ cells from E11.5 cKO-AGM or cKO-FL. CD45+ cells were sorted from E11.5 Ctr and cKO AGM and FL, followed by CFU assay. Colonies from E11.5 cKO AGM or FL were picked up and genotyped. Δ stands for excised allele. f stands for floxed allele. Genotyping analysis confirmed that all of these colonies exhibit incomplete excision of *Drosha* alleles.

Target Gene	miRNA	cKO (RPM)	Ctr (RPM)	Fold Change (cKO/Ctr)
Alox5	miR-19a-3p	0	6.097	0
	miR-125b-5p	223.524	371.701	0.60
	let-7a-5p	886.596	1499.464	0.59
	let-7c-5p	234.299	432.294	0.54
	let-7d-5p	15.0002	25.725	0.58
	let-7e-5p	472.823	1401.490	0.33
	let-7f-5p	1980.98	3018.423	0.65
	let-7g-5p	17.82	33.19	0.53
	let-7i-5p	541.80	1026.87	0.52
GATA2	miR-9-5p	593.670	1111.585	0.53
	miR-27a-3p	5.070	27.734	0.18
	miR-378a-3p	338.8779169	473.1924756	0.72
Runx1	miR-9-5p	593.6701662	1111.585297	0.53
	miR-27a-3p	5.070492522	27.73436468	0.18
	miR-30c-5p	59.26138135	113.4496294	0.52
	miR-126a-3p	210.6367102	364.2647535	0.58
	miR-126a-5p	1518.40124	2593.06261	0.59
	miR-181a-5p	1898.371273	2863.67364	0.66
	miR-181d-5p	199.5450078	276.4392653	0.72
	miR-199a-3p	248.3484983	302.2643802	0.82

Supplementary Table 4 List of miRNAs which target *Alox5, Runx1, or GATA2*. Small RNA sequencing result of ECs ($CD31^{+}CD45^{-}Kit^{-}$) from E11.5 Ctr or cKO embryos which are known to target Alox5, Runx1, or GATA2 are shown as average RPM (reads per million mapped reads). ND stands for “not detected”. MiRNAs indicated in bold are more than 40% reduction in cKO-ECs compared to Ctr-ECs (Fold Change of <0.6).

		Primer Sequence (5' to 3')
**Primers for Genotyping**	Drosha-F	GCA GAA AGT CTC CCA CTC CTA ACC TTC
	Drosha-R	CCA GGG GAA ATT AAA CGA GAC TCC
	Alox5-1	GCA GGA AGT GGC TAC TGT GGA
	Alox5-2	GCA GGA AGT GGC TAC TGT GGA
	Alox5-3	TGC AAC CCA GTA CTC ATC AAG
	Cre-F	TGC CAC GAC CAA GTG ACA GCA
	Cre-R	AGA GAC GGA AAT CCA TCG CTC
**Primers for qRT-PCR**	Alox5-F	GGCTGCAACCCAGTACTCAT
	Alox5-R	TTTGGTTGAGCTGGATGGCA
	ALOX5AP-F	CAGAATGCGTTCTTTGCCCA
	ALOX5AP-R	TCCTGCAGTCCAGAGTACCA
	CD31-F	TCA CCA TCA ACA GCA TCC AT
	CD31-R	GGT GCT GAG ACC TGC TTT TC
	Drosha-F	GATAGGAGCTGTTTACTTGGAGG
	Drosha-R	AGTTGCCGATCCGTATTTGG
	Blt1-F	GACTTGGCTGTGTTGCTCACTG
	Blt1-R	AGCAGGACACTGGCATACATGC
	Blt2-F	CACCTGTCACCTTCCTAAATCTC
	Blt2-R	GCCCTTCCAGCTCAGTAG
	Cysltr1-F	GGAAGTTTGTGAAAGGTGCTG
	Cysltr1-R	AGGAATGTCTGCTTGGTGC
	Cysltr2-F	GAATTTGGGAAAGGAAGAGTGAAG
	Cysltr2-R	ACATTTGCCGTACTCAGTCTC

Supplementary Table 5 List of primer sequences (5' to 3') for genotyping and qRT-PCR.
